# miRNeye: a microRNA expression atlas of the mouse eye

**DOI:** 10.1186/1471-2164-11-715

**Published:** 2010-12-20

**Authors:** Marianthi Karali, Ivana Peluso, Vincenzo A Gennarino, Marchesa Bilio, Roberta Verde, Giampiero Lago, Pascal Dollé, Sandro Banfi

**Affiliations:** 1 Telethon Institute for Genetics and Medicine, Via P. Castellino 111, 80131 Naples, Italy; 2 IGBMC (Institute de Génétique et de Biologie Moléculaire et Cellulaire); UMR 7104 CNRS; U 964 INSERM; BP 10142, 67404 Illkirch, France; 3 Department of Molecular and Human Genetics, Baylor College of Medicine, Houston, Texas 77030, USA; 4 ICB (Istituto di Chimica Biomolecolare), CNR, Via Campi Flegrei 34, 80078 Naples, Italy

## Abstract

**Background:**

MicroRNAs (miRNAs) are key regulators of biological processes. To define miRNA function in the eye, it is essential to determine a high-resolution profile of their spatial and temporal distribution.

**Results:**

In this report, we present the first comprehensive survey of miRNA expression in ocular tissues, using both microarray and RNA *in situ *hybridization (ISH) procedures. We initially determined the expression profiles of miRNAs in the retina, lens, cornea and retinal pigment epithelium of the adult mouse eye by microarray. Each tissue exhibited notably distinct miRNA enrichment patterns and cluster analysis identified groups of miRNAs that showed predominant expression in specific ocular tissues or combinations of them. Next, we performed RNA ISH for over 220 miRNAs, including those showing the highest expression levels by microarray, and generated a high-resolution expression atlas of miRNAs in the developing and adult wild-type mouse eye, which is accessible in the form of a publicly available web database. We found that 122 miRNAs displayed restricted expression domains in the eye at different developmental stages, with the majority of them expressed in one or more cell layers of the neural retina.

**Conclusions:**

This analysis revealed miRNAs with differential expression in ocular tissues and provided a detailed atlas of their tissue-specific distribution during development of the murine eye. The combination of the two approaches offers a valuable resource to decipher the contributions of specific miRNAs and miRNA clusters to the development of distinct ocular structures.

## Background

MiRNAs are a class of small non-coding RNAs that negatively regulate gene expression by binding to target sites in the 3' UTR of mRNAs. This binding affects the stability and translation of the target transcript [1,2]. To date more than six hundred highly-conserved miRNAs have been identified in the mouse genome (miRBase database, [3,4]) proposing miRNome as a new layer of gene regulation.

MiRNAs are key mediators of basic biological processes. In all plant and animal species examined, defects in miRNA function have profound effects on development [5,6]. In humans, deregulation of miRNA expression caused by mutations in either the miRNA or its target has been correlated with a number of clinically important diseases such as diabetes, neurodegenerative diseases and heart failure [7,8], among others. Moreover, there is growing evidence supporting a role of miRNAs in human cancers [9,10]. In this regard, miRNAs may constitute new targets of therapeutic interventions for a variety of diseases (reviewed in [11]). To gain insight into the functional role of miRNAs, both in physiological and pathological processes, it is essential to have precise information on their temporal and spatial expression profile. Recent adaptations in tools for expression and functional analysis, developed to counteract the limitations posed by the small size of miRNAs, have facilitated detection of the miRNA content of several tissues [12-14]. These studies revealed that about one third of miRNAs are expressed in a tissue-restricted manner in vertebrates [15-18].

The eye is a highly specialized organ whose development and function requires the precise coordination and timing of morphogenetic and cell differentiation events. Perturbation of miRNA function in the eye of conditional *Dicer *mouse mutants impairs the normal development of the retina, lens, cornea and optic chiasm [19-22]. Therefore, elucidation of the functional role of miRNAs is expected to provide key information to decipher the complex regulatory circuits underlying these processes. To define miRNA function in the eye, it is essential to obtain a high-resolution profile of their spatial and temporal distribution. This information, combined with *in silico *target prediction analysis, can also optimize the recognition of biologically significant mRNA targets. To date, only a limited number of reports are available on the expression profile of miRNAs in the mammalian eye. The majority of these publications analyze the miRNA content of the retina [18,23-28] while only a few address miRNA expression in non-neural parts of the eye [20,29,30]. Furthermore, examples of the cellular distribution of miRNAs in the eye are scarce, usually describing the spatial expression profiles of a limited number of miRNAs at a few developmental stages [31-35]. As a consequence, the information available on the complete set of miRNAs expressed in ocular tissues is only partial and fragmented.

To obtain a global view of the differential miRNA expression in the tissues that form the adult mouse eye, we performed microarray profiling of retina, retinal pigment epithelium (RPE), lens and cornea RNA. Cluster analysis allowed us to identify subgroups of miRNAs enriched in each of the above tissues. These miRNA clusters may reveal possible synergisms in the differentiation, cell-lineage commitment, development and maintenance of the analyzed eye structures. Moreover, to obtain a high-resolution atlas of the spatiotemporal distribution of these miRNAs in the eye, we performed RNA *in situ *hybridization (ISH) using Locked Nucleic Acid (LNA)-modified probes on sections of embryonic (E16.5), postnatal (P0, P8) and adult (P60) mouse eyes and implemented the data in a publicly available web database http://mirneye.tigem.it. Here, we describe the miRNA expression profiles in the main tissues of the eye and show the spatiotemporal distribution of 221 miRNAs. Finally, we propose a possible way on how cluster analysis of the miRNA differential expression can be exploited to advance our understanding of the functional role of selected miRNAs in a given tissue.

## Results and Discussion

### miRNAs show differential expression in murine eye tissues

We decided to define the complete repertoire of miRNAs that are expressed in any of four different eye tissues, *i.e*. retina, lens, cornea and RPE. As a first step, we collected retinas, lenses, corneas, RPE tissues and entire eyes from 8-week old C57BL/6 mice. We chose to use the C57BL/6 strain that has a pigmented RPE in order to limit the carry-over contamination of the retina samples with RPE cells during tissue dissection. Following total RNA extraction from these eye tissues, we carried out miRNA profiling using the miRCURY LNA™arrays provided by Exiqon (see Methods). Total RNA from entire eyes was included as a reference to determine differential expression. The miRNAs that showed, in one or more tissues, a difference in Log2 median ratios (ΔLMR) equal or superior to 0.5 (n = 285 out of 597 analyzed) compared to the 'entire eye' reference were considered for further analysis.

**Figure 1:**
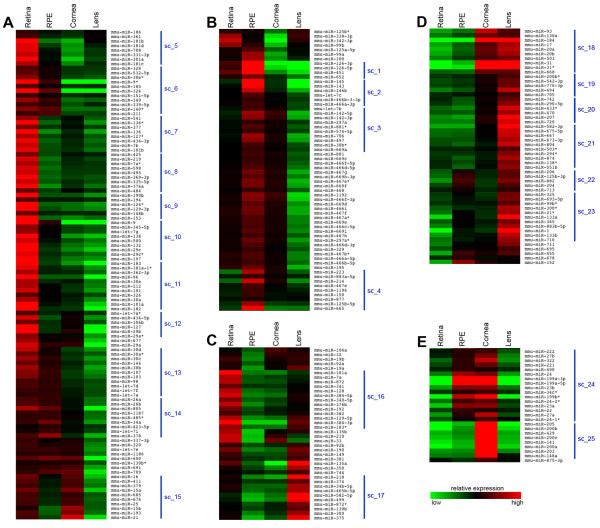
**Differential expression patterns of miRNA clusters in murine ocular tissues**. Heat-map diagram of the two-way hierarchical clustering of 285 miRNAs (Delta Log2 median ratio ΔLMR ≥ 0.5) showing relative miRNA expression levels in different tissues of the adult murine eye. Colors indicate relative expression compared to the mean for each miRNA in the entire eye. Distinct clusters of tissue-enriched miRNAs are revealed. At a five-node-level, five miRNA clusters are obtained enriched in one or more tissues: retina (A), RPE (B), retina and/or lens (C), lens and/or cornea (D) and cornea and/or RPE (E). The 25 miRNA sub-clusters selected for GO term enrichment analysis are highlighted with blue lines. From the following groups of miRNAs with identical sequence, only the first member is annotated in the heat-map: mmu-let-7a*/mmu-let-7c-2*; mmu-miR-466a-3p/mmu-miR-466b-3p/mmu-miR-466c-3p/mmu-miR-466e-3p; mmu-miR-467a*/mmu-miR-467d*; mmu-miR-297a*/mmu-miR-297c*/mmu-miR-297b-3p and mmu-miR-199a-3p/mmu-miR-199b. sc: sub-cluster.

To demonstrate whether miRNAs show tissue-enriched expression in a specific eye compartment, the 285 miRNAs that passed the above threshold were used in a two-way hierarchical clustering analysis. This analysis revealed distinct miRNA clusters that are differentially expressed in one or more of the tissues analyzed (Additional file 1). The resulting dendrogram could be divided in five main clusters (A-E) (Figure 1A-E; Green brackets in Additional file 1) enriched in miRNAs predominantly expressed in retina (Cluster A; Figure 1A), RPE (Cluster B; Figure 1B), retina and lens (Cluster C; Figure 1C), lens and cornea (Cluster D; Figure 1D), and cornea and RPE (Cluster E; Figure 1E). Within these five clusters, we defined 25 smaller groups (sub-clusters) of miRNAs with highly similar expression profiles (Blue lines in Figure 1A-E and Additional file 1). The reliability of the clustering analysis of microarray data was confirmed by assessing the overall relationships among the analyzed tissues, which were in line with their developmental origin (Additional file 1B). The significant number of miRNAs that show differential expression profiles among the ocular tissues and their organization in distinct clusters suggest that the regulation of eye development and function by miRNAs requires an intricate biological network. Our microarray results are overall concordant with similar global expression studies on single eye tissues previously reported in mouse or other vertebrates (reviewed in [36]). However, this is the first example of a simultaneous analysis of the main ocular structures, which therefore allows a direct semi-quantitative assessment of the differential miRNA expression levels in the major components of the mouse eye.

To identify whether groups of miRNAs with comparable expression profiles may be involved in the regulation of specific biological functions, we performed an *in silico *functional annotation analysis of their predicted target genes using the 25 above- described co-expression sub-clusters (Blue lines in Figure 1A-E and Additional file 1). For each miRNA present within these sub-clusters, we compiled a non-redundant list of predicted targets combining the output of miRanda, TargetScan and Pictar [37-39], three commonly used miRNA target prediction software. Subsequently, we performed a Gene Ontology (restricted to Biological Processes) and KEGG pathway annotation analysis for the target lists of each single miRNA, as described in Methods. We then compared the annotation term lists of all predicted target genes for each miRNA belonging to each sub-cluster and counted the occurrence of shared individual GO/KEGG terms (Additional file 2). An arbitrary cut-off value of 70% was set and only terms enriched in the predicted target gene lists of at least 70% of the miRNAs within a cluster were retained (see Additional file 3). As a first result, we found a significant enrichment in several GO/KEGG terms related to neuronal development and function (Additional files 2 and 3), which, considering the neural origin of the eye, is a good indication of the reliability of our analysis. The above-mentioned enrichment in neuron-related terms was more prominent in sub-clusters that contained miRNAs strongly expressed in retina (Additional files 2 and 3). Another interesting example was sub-cluster 24, strongly expressed in cornea and RPE, which showed enrichment for the GO term 'epithelium development' (Additional files 2 and 3). Overall, we found enrichment for specific processes, consistent with the physiology and function of the ocular tissues in which the analyzed miRNAs were predominantly expressed. This suggests that the above-described cluster analysis of miRNA expression data, although requiring further experimental validation, can be used to gain insight into the specific functional processes regulated by miRNA-target networks within the eye.

### miRNAs show distinct spatiotemporal distribution during eye development

**Figure 2:**
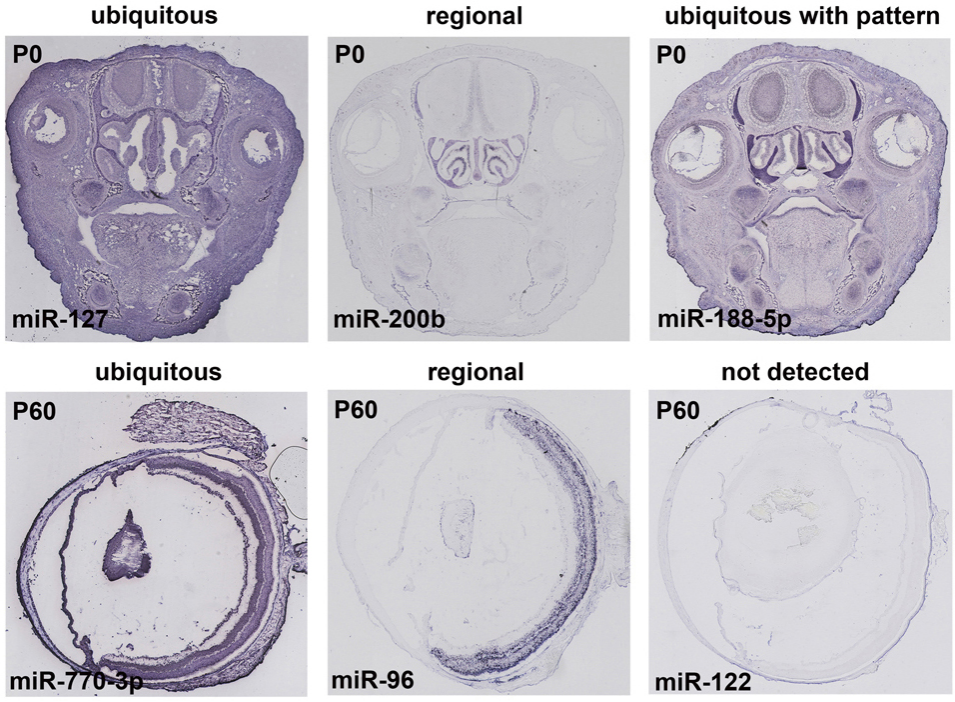
**Examples of the *in situ *hybridization signal distribution per developmental stage**. Examples of expression patterns classified as 'ubiquitous', 'ubiquitous with pattern', 'regional' and 'not detected' in sections of newborn heads (upper panel) and/or adult eyes (lower panel).

To study the spatiotemporal distribution of the above-mentioned miRNAs at the cellular level, we performed RNA ISH profiling on sections of murine eyes at different developmental stages. To this purpose, we carried out high-throughput robotic RNA ISH on serial sections of whole heads and eyes (at postnatal stages) using LNA-modified DNA oligonucleotide probes (Figure 2). The ISH analysis was performed in the albino CD1 wild-type mouse strain in order to enable signal detection in the non-pigmented RPE. The selection of miRNAs to analyze mostly relied on the evaluation of microarray profiling data described above. We prioritized for the analysis 212 miRNAs that displayed the highest levels of differential expression in the adult eye by microarray. Moreover, we selected 36 miRNAs that were not detected in a reliable manner by microarray. Given the different resolution and sensitivity of the two technologies, we decided to analyze also the latter ones to verify the ability of RNA ISH to detect miRNAs expressed at very low or single-cell levels. Indeed, the ISH protocol we used includes a signal amplification step which can reveal low abundance transcripts that would have been otherwise undetectable [40]. As a final result, taking also into account technical failures and probe availability, we obtained reliable ISH data for 221 miRNAs (Additional file 4).

For this study, we looked at time points that correspond to milestones of development and differentiation of the major eye structures. In particular, we analyzed miRNA expression at a prenatal stage (E16.5), when the major structures of the eye are already formed, although not fully differentiated; at the date of birth, postnatal day (P) 0, when the newborn mouse eye is still developmentally immature; at P8, when the majority of retinal cells have exited the cell-cycle and occupy their final positions within the retina and, finally, at the P60 stage that corresponds to full adulthood and maturation of the eye is complete. The expression patterns obtained were annotated based on the overall signal distribution (Figure 2) and on the anatomical structures and/or cell-types that stained positive for miRNA expression. When a uniform signal was visible across the entire section, the pattern was defined 'ubiquitous' (ubi) (*e.g*. miR-127 in P0 head and miR-770-3p in P60 eye; Figure 2). Whenever the signal was restricted to specific cells or structures the expression pattern was defined 'regional' (reg) (*e.g*. miR-200b staining of the olfactory epithelium at P0 and miR-96 specific staining of the adult retina; Figure 2), while in the absence of a visible signal, the attribute 'not detected' (ND) was assigned (*e.g*. miR-122 in P60 eye; Figure 2). Finally, an additional category, 'ubiquitous with pattern' (uwp), was defined for head sections at E16.5 and P0 in cases where a signal was detected in most cells with some structures showing stronger staining (*e.g*. miR-188-5p in P0 head; Figure 2).

Overall, we identified a range of different spatially and/or temporally restricted expression profiles for 178 miRNAs, while 43 did not yield any signal in the eye at any developmental stage. We found a number of miRNAs (n = 30) that despite displaying significant differential expression by microarray, were not detected by RNA ISH. This could be explained either by differences in sensitivity between the two approaches or by possible miRNA expression level differences across mouse strains. On the other hand, by RNA ISH we obtained a clear regional hybridization signal for 14 miRNAs that were not detected in a reliable manner by microarray profiling (*e.g*. miR-188-5p, miR-296-5p, miR-680, miR-681; see database). These observations highlight the advantage of employing diverse approaches in determining miRNA expression profiles. A summary of the ISH signal distributions observed at each developmental stage is shown in Table 1. A detailed annotation of the expression patterns at all time points is listed in Additional file 4. Although broadly in agreement with previously published work, in some cases the ISH images revealed differences in miRNA distribution (*e.g*. miR-182). We believe that these discrepancies could be attributed to differences in the experimental conditions and, most importantly, to the improved sensitivity in the detection of low-abundance transcripts offered by the signal amplification step of the automated approach.

**Table 1:** Occurrence of the different types of ISH signal distribution across development.

Developmental Stage	Ubiquitous	Ubiquitous with pattern	Regional	Not detected
E16.5	53	58	37	73
P0	30	89	42	60
P8	87	NA*	71	63
P60	51	NA*	106	64

* NA = non applicable

**Figure 3:**
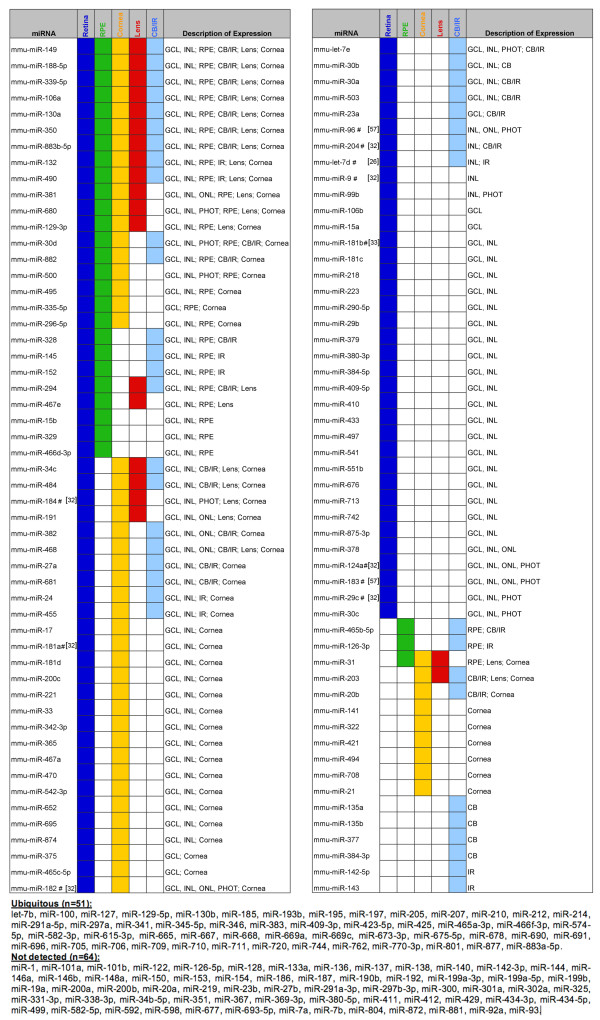
**Graphical summary of miRNA expression patterns in the adult mouse eye**. This catalog summarizes miRNA localization in the retinal cell layers and ocular tissues analyzed at P60. miRNAs that were ubiquitously distributed or did not show any detectable expression are listed in the footnote. The regional miRNAs that had been previously reported to be expressed in the vertebrate eye by ISH are indicated with a # sign and an appropriate reference. Abbreviations: CB = Ciliary body; GCL = Ganglion Cell Layer; INL = Inner Nuclear Layer; IR = Iris; ONL = Outer Nuclear Layer; PHOT = Photoreceptors; RPE = Retinal Pigment Epithelium.

The ISH data from the adult eye could be directly compared to the microarray profiling performed at the same stage (see above and Table 2). A summary of miRNA distribution in the different anatomical structures of the adult eye as well as in the retinal cell layers is shown in Figure 3. Overall, the comparison between the RNA ISH annotation and the microarray data revealed a high degree of consistency and the combination of the two datasets largely contributes to the definition of the tissue distribution of miRNAs in the eye.

**Table 2:** Overlap between the microarray and ISH data.

		***in situ hybridization***		
		**regional**	**not** **regional**^**1**^	**Total**
	
	**diff. expressed^2^**	92	93	**185**
***Microarray***				
	**not diff. expressed^3^**	14	22	**36**
	
	**Total**	**106**	**115**	**221^4^**

^1^: miRNAs that were annotated as 'ubiquitous' or 'not detected'

^2^: miRNAs with ΔLMR ≥ 0.5

^3^: miRNAs labeled as NA in at least one of the nine samples analyzed

^4^: Total number of miRNAs analyzed both by microarray and ISH

### A web atlas of miRNA expression in eye structures

To make these data readily accessible we constructed miRNeye, a web-searchable atlas of miRNA expression patterns in the mouse eye that can be accessed at http://mirneye.tigem.it. The atlas offers high-resolution images of the cellular distribution of miRNAs in the mammalian eye and represents a useful tool in the study of miRNA contribution in eye formation, maintenance and function. Combined with target prediction software, knowledge of the miRNA cellular distribution, as well as of the miRNA clusters of co-expression, can greatly aid in unraveling their role in the eye. Below we provide a more detailed description of the spatiotemporal miRNA expression profiles we detected in each of the analyzed murine eye tissues.

### miRNAs show cell-type enriched expression in the retina

**Figure 4:**
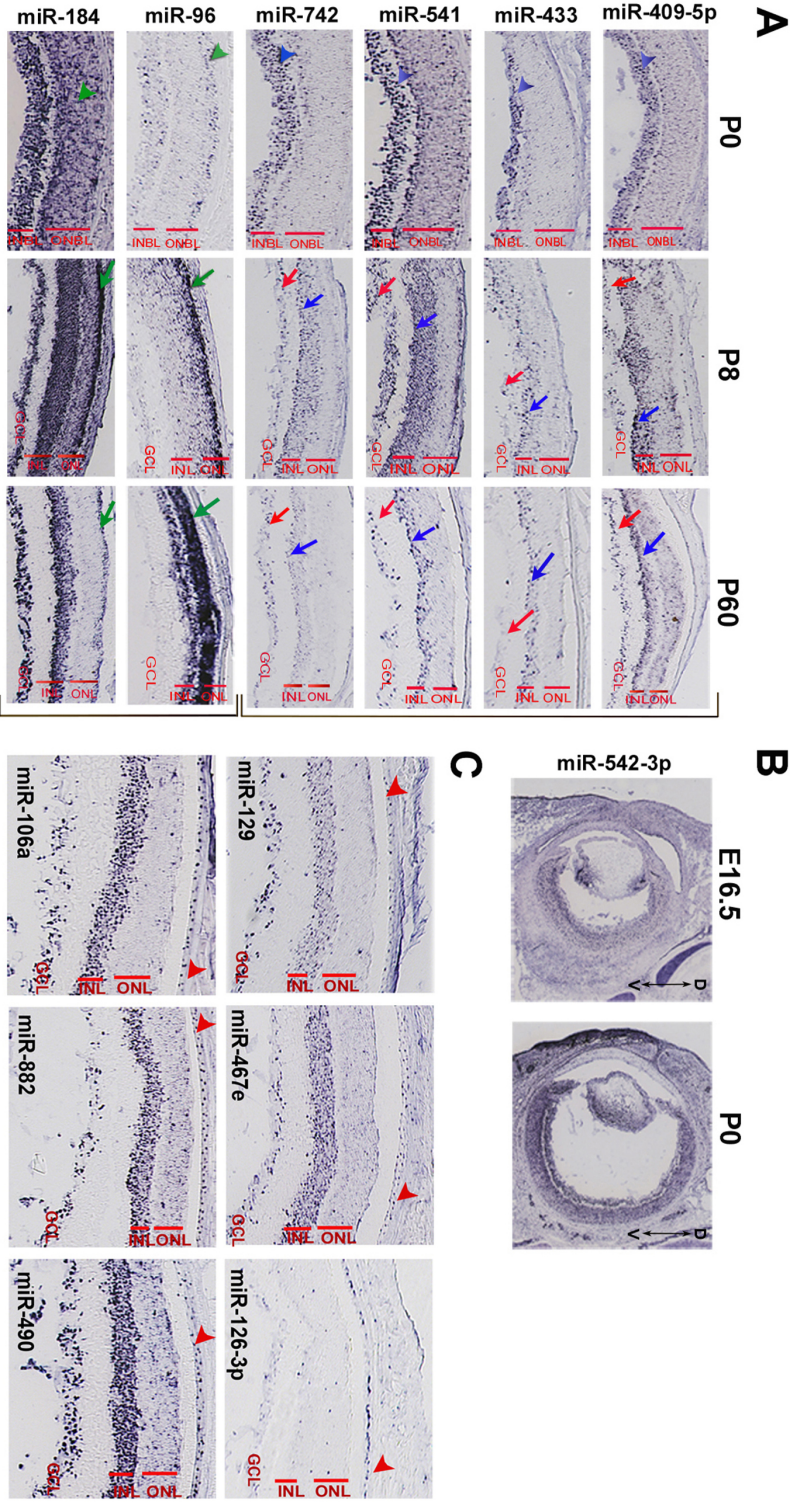
**Examples of miRNAs expressed in the retina and the retinal pigment epithelium**. (A): Representative examples of possible amacrine-expressed (top bracket) and photoreceptor-expressed (lower bracket) miRNAs. Most of the retinal patterns are already detectable at E16.5 and P0, often with a specificity that is maintained throughout life. For miR-409-5p, miR-433, miR-541 and miR-742 (top bracket; panel A) a weak staining is detectable in the inner neuroblastic layer (INBL; blue arrowheads) at P0. At P8 and P60, these miRNAs are expressed in the ganglion cell layer (GCL; red arrows) and the inner nuclear layer (INL; blue arrows), with stronger expression in the vitreal part of the latter where amacrine cells reside. The retinal distribution of the above miRNAs had not been previously described. miR-96 and miR-184 (lower bracket; panel A) are examples of miRNAs that stain the outer nuclear layer (ONL; green arrows) and/or the photoreceptors at P8 and P60 while at P0 they are detected in the outer neuroblastic layer (ONBL; green arrowheads). (B): miR-542-3p shows an apparent low-dorsal to high-ventral gradient expression in the developing retina at E16.5 and P0. (C): Representative examples of miRNAs expressed in the retinal pigment epithelium (RPE). Red arrowheads indicate the RPE monolayer.

We found that the majority of miRNAs showing, by RNA ISH, regional distribution in the adult eye (89 out of 106) were detected in the retina, in one or more of its cell layers. Among them, we both found miRNAs with previously reported retinal expression domains *e.g*. miR-124a, miR-29c, miR-9, miR-182, miR-181a [32], among others, but we also identified at least 70 miRNAs, which, to our knowledge, were not previously described to have a regional expression in the retina in any vertebrate species (Figures 3 and 4).

Most of the miRNA expression patterns in the retina had a developmental onset. At E16.5, retina-expressed miRNAs stained mainly the inner neuroblastic layer (INBL) where newborn ganglion cell neurons are located (see Additional file 4 and miRNeye database). Likewise, in retinas of newborn mice (P0), miRNA expression was mainly detected in the vitreal cell layer of the retina (*i.e*. INBL) where differentiated ganglion and amacrine cells reside (see blue arrowheads in Figure 4A, first column). We did not encounter any case in which miRNA expression was detected only in the mitotic progenitors of the outer neuroblastic layer (ONBL) at either E16.5 or at P0. This was expected given that the miRNAs we analyzed by RNA ISH were selected based on a microarray study carried out on fully-differentiated, adult ocular tissues and is also in line with the general view that miRNAs principally promote cell differentiation [15]. In a limited number of cases (*e.g*. miR-96, miR-184), staining at P0 was extended also to the ONBL, where immature photoreceptors are found (see green arrowheads in Figure 4A). For these miRNAs, photoreceptor-enriched expression was further confirmed at P8 and P60 (green arrows in Figure 4A, second and third column). Interestingly, among the retina-expressed miRNAs, we identified miR-542-3p, that showed an apparent low dorsal to high ventral gradient of expression in the developing (E16.5) and newborn (P0) retina (Figure 4B).

The laminar organization of the mammalian retina - in particular at the adult stage when all differentiation events have been completed - can be exploited to deduce the possible identity of cells expressing a given miRNA. On that basis, we identified miRNAs (*e.g*. miR-409-5p, miR-433, miR-541, miR-742) that, in the adult retina, are clearly detected in the vitreal part of the Inner Nuclear Layer (INL) and are likely to be expressed in amacrine cells (blue arrows in the third column of Figure 4A; Database). Another class of miRNAs (*e.g*. miR-29c, miR-30d, miR-96, miR-99b, miR-124a, miR-182, miR-183, miR-184, miR-381, miR-425) also stained, in the postnatal retina, the Outer Nuclear Layer (ONL) where rod and cone photoreceptors reside (green arrows in the third column of Figure 4A; Database). Many of the latter miRNAs were also expressed at P0 in the olfactory epithelium, which, similarly to the retina, is equipped with sensory epithelia (see database). Notably, the vast majority of the regional miRNAs detected in the retina stained preferentially, though to a different extent, the Ganglion Cell Layer (GCL) and the Inner Nuclear Layer (INL) and to a much lesser extent the Outer Nuclear Layer (ONL). This is consistent with the above-described preferential staining of the INBL at E16.5 and P0 and suggests that the neuronal populations that are part of the innermost retinal cell layers need, for their proper function, the expression of a wider catalog of miRNAs compared to photoreceptors cells. Finally, we observed subtle variations in the spatiotemporal expression patterns of polycistronically transcribed miRNAs *(e.g*. the miR-183/96/182). We believe that they could be explained either by the differential processing of the primary transcript or by the differential affinity of the probes to their respective mature miRNA.

### miRNAs expressed in the retinal pigment epithelium

The RPE is a monolayer of pigmented epithelium that lies between the photoreceptors of the neural retina and the choroid. Similarly to the neural retina, the RPE derives from the anterior neural plate (neuroectoderm). Exogenous signals from the surrounding periocular mesenchyme induce RPE specification whereas inputs from the surface ectoderm drive differentiation of the prospective neural retina (reviewed in [41]). The initial regionalization between the presumptive RPE and neural retina is further consolidated by the expression of distinct sets of regulators, including well-established transcription factors and possibly tissue-specific miRNAs [42].

By ISH we detected 29 miRNAs in the adult RPE (examples in Figure 4C, arrowheads), 26 of which also stained the neural retina. Instead, from the microarray analysis, we identified 68 miRNAs that are enriched in the RPE. By hierarchical clustering the majority of these miRNAs grouped in cluster B (Figure 1B) and some of them were found to share expression enrichment both in RPE and retina, similar to what we observed by ISH. This similarity in the miRNA content of RPE and neural retinal cells could be attributed to the common origin of the two tissues. Instead, the miRNAs that are differentially expressed between RPE and retina could be those that principally contribute in the establishment and maintenance of RPE identity. Finally, miRNAs that were concurrently enriched in RPE, cornea and/or lens (Figure 1B, D, E) may include miRNA signatures associated with epithelial physiology.

### miRNAs expressed in the cornea

The cornea is an avascular, transparent structure within the anterior eye, of prime importance for light refraction and vision. It consists of three distinct layers: the epithelium, the stroma and the endothelium. The epithelium derives from the surface ectoderm whereas the corneal stroma and endothelium are formed by neural crest and mesoderm-derived mesenchymal cells [43]. Corneal function relies on the self-renewal capacity of the epithelium which in turn is ensured by a population of Limbal Epithelial Stem Cells (LESCs) mostly present in the limbus, which is located at the corneo-scleral junction [44,45]. To date, little is known about the miRNA content of the cornea and on its contribution to corneal differentiation and homeostasis.

**Figure 5:**
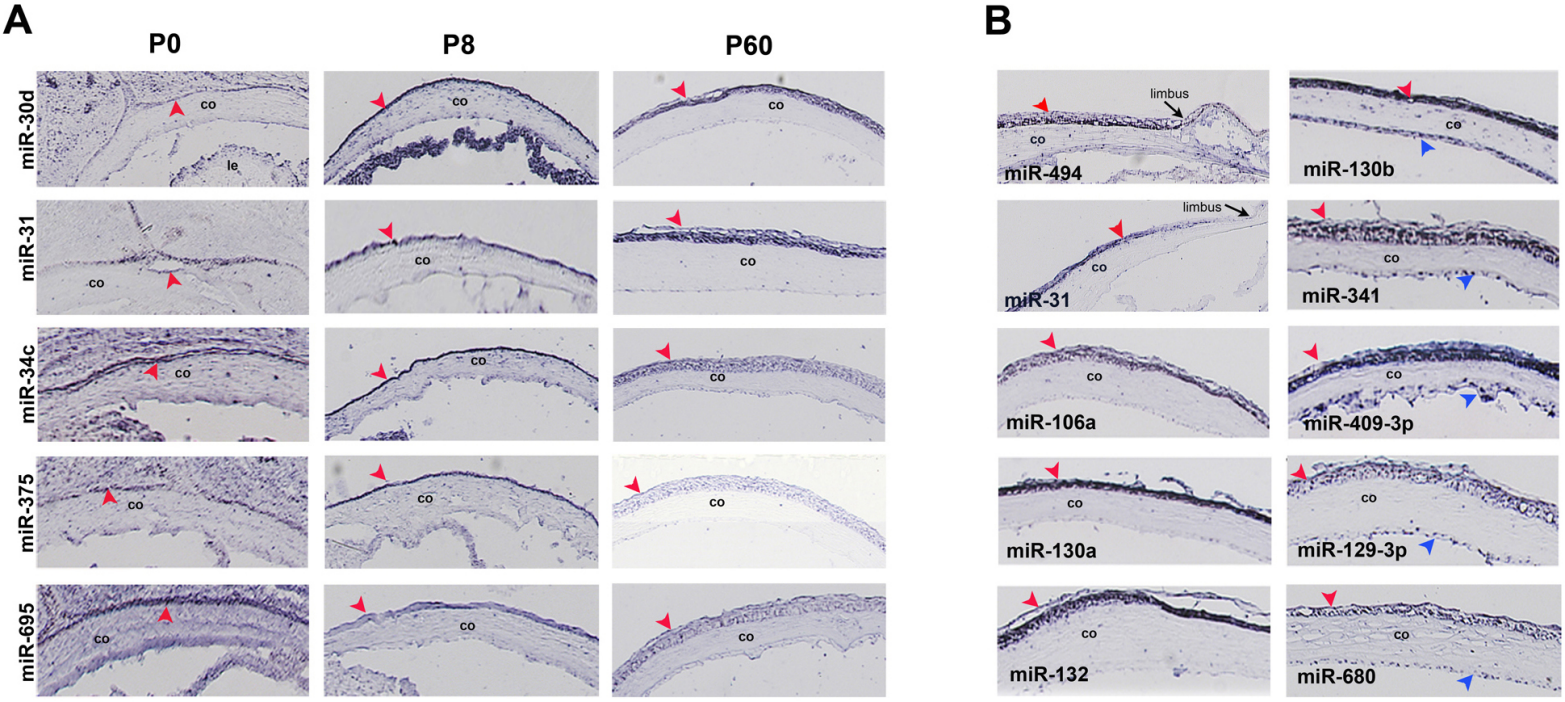
**Expression patterns of miRNAs detected in the cornea by ISH**. (A): Representative examples of miRNA expression that could be detected in the cornea (red arrowheads) already at P0. Expression in the corneal epithelium persisted throughout life and was detected also at P8 and P60. (B): Two basic patterns of miRNA expression were observed in the adult cornea. For some miRNAs (examples in left column) corneal expression was restricted in the epithelium (red arrowheads), while for others (examples in right column) it was detected both in the epithelium (red arrowheads) and endothelium (blue arrowheads). miR-31 was not detected in the limbal region (limbus, arrow). Abbreviations: co = Cornea.

By RNA ISH, miRNA expression in the cornea could be detected as early as E16.5 and P0 in 5 and 14 cases, respectively (Figure 5A; Additional file 4; Database). At P8, when the epithelium is actively proliferating and at P60, when the cornea has reached adult thickness, we could detect 22 and 54 regional miRNAs, respectively (Figure 5A; Additional file 4; Database). From the latter group, 11 stained clearly both the corneal epithelium (red arrowheads) and endothelium (blue arrowheads, right column in Figure 5B; Database) while the remaining ones (*e.g*. miR-106a, miR-130a, miR-132, miR-494, miR-31) were detected only in the epithelium (left column in Figure 5B; Database). Collectively, our results confirm the cornea expression of miRNAs already reported in literature [27,31,32] and reveal the corneal-enrichment of others that had not been previously described to be expressed in the eye (*e.g*. miR-130a, miR-130b, miR-132, miR-129-3p, the miR-200 family, miR-468, miR-874). Most importantly, the ISH analysis enhances the current scarce knowledge on the cellular distribution of these miRNAs within the cornea. In particular, we can differentiate between miRNAs that are expressed exclusively in the ectoderm-derived epithelium and others that are expressed both in the epithelium and the endothelium. Interestingly, among the miRNAs detected in the corneal epithelium, we could highlight different patterns of distribution in the limbus. For instance, 14 miRNAs clearly stained the entire corneal epithelium, including the limbus (*e.g*. miR-494; Figure 5B; Database), while at least 9 were not detected in the limbal region despite being expressed in most of the corneal epithelium (*e.g*. miR-31, Figure 5B; Database). Thus, it is tempting to speculate that such differences in miRNA expression within the limbus may contribute to the establishment of the proper environment required for LESCs maintenance.

### miRNAs expressed in the lens

The lens is an elastic, transparent structure located in the anterior segment of the eye with a role in light refraction and focal distance adjustment. During embryogenesis, the lens derives from the surface ectoderm following inductions from the neuroectoderm and consists of the capsule, a layer of cuboidal epithelium and the lens fibers.

**Figure 6:**
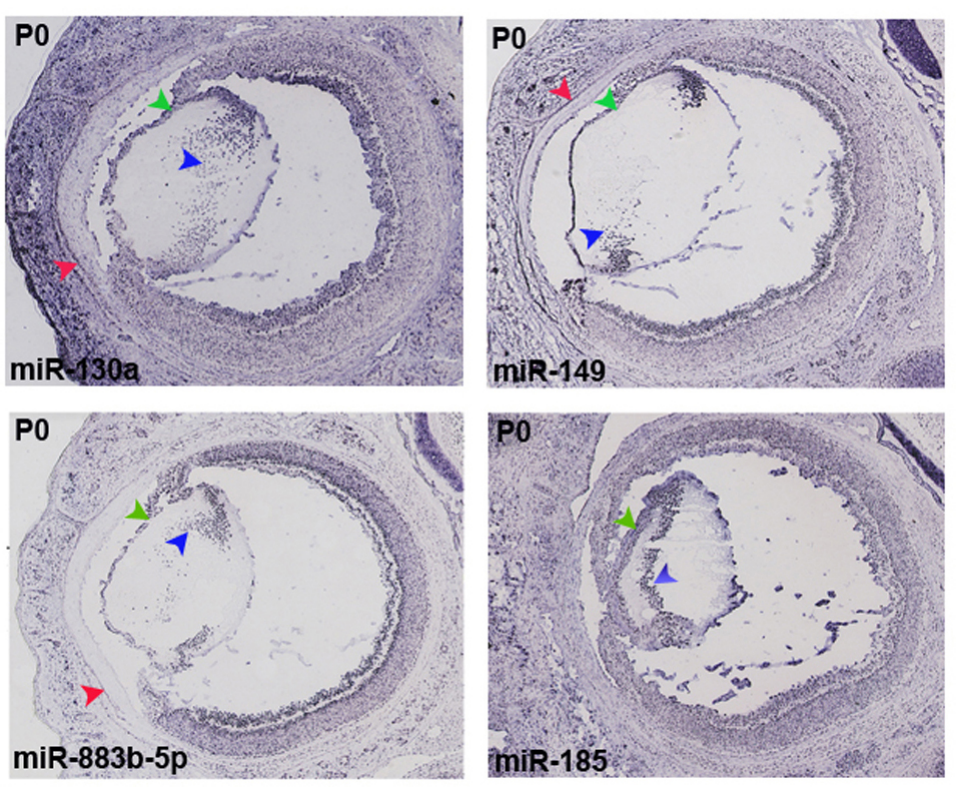
**Expression patterns of miRNAs detected in the lens by ISH**. Representative examples of miRNA expression detected in lens epithelium (green arrowheads) and in the equatorial zone (blue arrowheads) on sections of P0 eyes. For miR-130a, miR-149 and miR-883b-5p concomitant staining of the corneal epithelium can be seen (red arrowheads).

By RNA ISH we detected 39 regional miRNAs in lens, especially at prenatal (E16.5) and postnatal (P0) stages when the lens structure remains intact after sectioning. In all cases, staining was evident in the anterior lens epithelium, where cells actively proliferate during lifetime and in the equatorial zone, where cells differentiate to lens fiber cells (Figure 6). Interestingly, some of the lens-enriched miRNAs are expressed at significant levels in the cornea (Figure 1D) and by ISH we frequently observe concurrent lens and cornea miRNA expression (*e.g*. miR-130a, miR-149, miR-883b-5p; red arrowheads in Figure 6), probably reflecting the common origin of these epithelial tissues.

### miRNAs detected in other ocular structure

**Figure 7:**
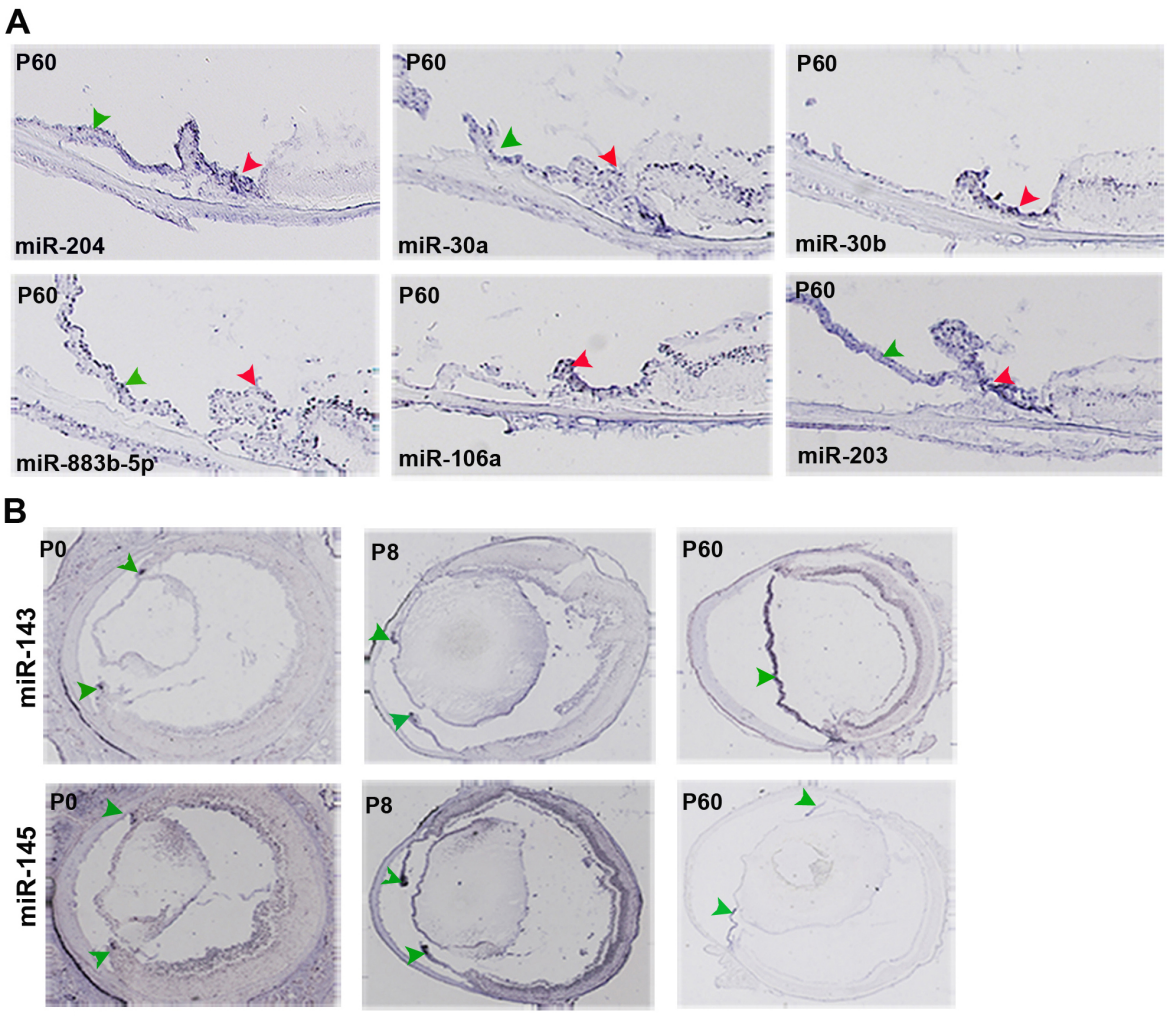
**miRNAs detected in the ciliary body and iris**. (A): Examples of miRNAs detected in the ciliary body (red arrowhead) and iris (green arrowheads) at P60. (B): Two examples of iris-specific miRNA expression patterns conserved across development. miR-143 and miR-145 are specifically detected in the iris at P0, P8 and P60 (green arrowheads).

RNA ISH analysis revealed a number (n = 50) of regional miRNAs that stained specifically the uveal tunics of the mouse eye in particular the iris and the ciliary body (CB) (Additional file 4; Figure 7A). Among these, there were miRNAs with a reported role in smooth muscle (*e.g*. miR-143 and miR-145) [46] and stem cell differentiation (*e.g*. miR-106a and miR-203) [47,48] (Figure 7A, B). This is in line with the fact that both the iris and ciliary body consist mainly of smooth muscle while their pigmented epithelia are niches of progenitor cells with stem-like properties of self-renewal and multi-potentiality [49,50]. The remarkable plasticity of these cell populations allows them, under appropriate culture conditions, to be trans-differentiated into pigmented neurospheres that express neuronal or glial markers [50]. In that respect, the miRNAs we detected in the iris and ciliary body (Additional file 4; Database) could represent important mediators of stemness and plasticity of pluripotent cells with neurogenic potential. This is particularly true for those that will turn out to be specifically expressed in the pigmented epithelia of these structures, though further experimental validation will be needed to confirm this hypothesis. Similarly, the strong and specific staining for miR-143 and miR-145 in the iris sphincter at all developmental stages analyzed (Figure 7B) is consistent with recent reports that demonstrate the key role of these two miRNAs in modulating smooth muscle cell proliferation and differentiation [46]. By microarray profiling these two miRNAs are highly enriched in the RPE (Figure 1B). That could in part explain the potential of RPE cells to lose epithelial characteristics and transdifferentiate into myofibroblasts during pathogenesis of proliferative vitreoretinopathy [51].

## Conclusions

As miRNAs turn out to represent crucial regulatory elements, sharing common principles with transcription factors, it is fundamental to establish their distribution and decipher their functional role in the eye in order to obtain a complete picture of the gene networks operating therein in health and disease states. Over the past few years, several miRNAs have been shown to be strongly and specifically expressed in the eye, suggesting that these small molecules play indispensable regulatory roles in eye biology. However, most of the previously published reports focused on the analysis of the miRNA component of the retina. Considerably less is known on the miRNA content of the RPE and of the non-retinal parts of the eye such as the lens and cornea. In addition, there is no report of a systematic analysis of miRNA expression in the eye at a cell-specific resolution achieved by RNA ISH on tissue sections. Hence, the work described here provides a significant contribution to the expansion of our knowledge both in the field of eye and miRNA biology. This is the first atlas of the eye miRNome that offers the possibility to explore miRNA expression profiles in the various ocular structures and cell-types during mouse development. The amplification protocol of the ISH procedure we used greatly enhanced the sensitivity of signal detection revealing novel, previously overlooked miRNA patterns. In particular, we analyzed the cellular distribution in the eye of 221 miRNAs and demonstrated a regional localization for 122 of them.

Furthermore, the parallel miRNA array analysis of the retina, RPE, cornea and lens allows a direct and semi-quantitative comparison of the miRNA content across the main ocular tissues and complements the qualitative data on miRNA localization obtained by RNA ISH. In particular, our approach highlights that eye development, maintenance and function is accompanied by the coordinated expression of a large set of miRNAs, which display distinct and overlapping patterns among the various tissues of the eye. As miRNAs are proposed to act mainly as fine-tuners of the transcriptome (reviewed in [52]), identifying groups of miRNAs that are expressed in a given tissue offers a precious resource in evaluating targets and pathways that direct the differentiation and maintenance of the ocular structures. By combining techniques with different sensitivity and data output we could identify distinct miRNA clusters that show comparable expression patterns in the eye. We coupled this information with target prediction and performed GO term functional analysis in an attempt to shed light to the complex miRNA regulatory networks that operate during eye development and function. The analysis revealed that miRNAs with highly correlated expression profiles may contribute, via their targets, to common pathways and cellular processes that are characteristic of the tissue in which these miRNAs are predominantly expressed.

Finally, deciphering the expression patterns of miRNAs is not only an important step towards a better comprehension of the molecular mechanisms that underlie eye function but can possibly aid the elucidation of the molecular basis of eye disease. Although a direct involvement of miRNAs in the pathogenesis of eye diseases has not yet been demonstrated, the latter hypothesis is extremely likely based on recent reports demonstrating that even alterations of a single miRNA can be responsible for human genetic diseases [53]. In that respect, precise knowledge on the sites and timing of miRNA expression in the eye will allow to evaluate whether modulation of expression levels and/or cellular distribution of specific miRNAs could constitute a means of therapeutic intervention in certain eye diseases.

Overall, the described miRNA atlas offers a much-needed, extensive and dynamic view on the miRNA inventory of the eye during development. This information will provide the foundation for a better understanding of the miRNA-mediated regulation of specific developmental pathways in the eye with eventual implications in diagnostics and therapy.

## Methods

### Sample Preparation

Total RNA from eye tissues of 8-week old adult C57BL/6 mouse was obtained using the miRNeasy kit (Qiagen) according to the manufacturer's instructions. RNA was quantified using the NanoDrop 1000 (Thermo Fisher). RNA quality was assessed by gel electrophoresis.

For the ISH experiments, CD1 embryos were removed by Caesarean section at the developmental stage of E16.5. Heads were surgically removed from E16.5 and P0 mice. P8 and P60 mice were sacrificed by cervical dislocation and the eyes were surgically removed and marked with a burn spot in order to define the dorso-ventral orientation. The optimal conditions for sample pre-treatment prior to automated ISH with LNA probes were set-up in preliminary test experiments. All specimens were embedded in O.C.T. compound (Tissue Tek) and cryosectioned. Serial frontal 18 μm cryostat sections were systematically collected on microscope slides starting at a standardized plane of section to ensure reproducible anatomical coverage. The sections were fixed in 4% paraformaldehyde in PBS, acetylated in 1 × triethanolamine and dried in an ethanol series.

All animal manipulations were done in accordance with the European regulations for the use of laboratory animals and authors confirm adherence to the ARVO (Association of Research for Vision and Ophthalmology) Statement for the Use of Animals in Ophthalmic and Vision Research.

### Microarray experiment

Microarray-based miRNA profiling of total RNA isolated from eye tissues was performed by Exiqon on the miRCURY LNA™Array Version 11.0 annotated to miRBase 12.0 applying a "common reference" design in which each sample in the study is hybridized against a common reference. Microarray hybridizations were performed in duplicates. Briefly, biological duplicates of each tissue (*i.e*. Retina, Lens, Cornea, RPE; labeled with Hy3) and RNA from entire eyes (labeled with Hy5) were hybridized on the same slide. To determine dye-bias, the set-up included an additional slide on which RNA from the entire eye was labeled with both dyes. All samples were then normalized against the common reference allowing direct comparison among them. The data obtained was normalized using a global LOESS (Locally Weighted Scatterplot Smoothing) regression algorithm [54]. The microarray analysis was performed according to MIAME standards and the results are available from the GEO database with the accession number GSE22882. MiRNA expression of each sample was compared to the reference RNA ('entire eye') and the difference in Log2 median ratios (ΔLMR) was determined (see Additional file 5 for a complete list of differential expression values). MiRNAs lacking reliable read-outs were labeled with the acronym "NA" (*e.g*. when 3 or more of the 4 replicated measures were flagged by the image analysis software due to the presence of empty spots, saturated spots or spots lacking optimal morphology, or when the normalized signal intensities were comparable to the background level, set as 1.5× the median signal intensity of the given slide). A false discovery rate (FDR) <0.05 was used to assess significant miRNA differential expression (estimated by 1000 permutations and calculated with the freeware dCHIP; http://biosun1.harvard.edu/complab/dchip/).

### Cluster Analysis

Cluster analysis was performed in Java using the MultiExperiment Viewer (MEV) packages ver. 4.5 [55]. An average linkage clustering analysis of the data was based on Pearson Correlation distance.

### Gene Ontology analysis

Gene Ontology (GO) analyses were performed using the web-tool DAVID at http://david.abcc.ncifcrf.gov/home.jsp and default parameters [56]. GO analyses were performed on the non-redundant list of predicted targets compiled from three target prediction software (MiRanda, Pictar, TargetScan) [37-39]. The obtained BP and KEGG categories were filtered for FDR ≤ 5 and FDR ≤ 20, respectively, against the default *Mus musculus *background (DAVID 6.7). Redundant terms and commonly encountered categories were eliminated. Results are shown in Additional file 2.

### RNA *in situ *hybridization and Image Acquisition

Hybridizations were performed in an automated manner using a Tecan Genesis liquid handling platform [40]. Briefly, the slides were placed in flow-through chambers positioned into a temperature-controlled rack. All solutions were then added using a computer-controlled liquid handling system. This system allowed the concurrent use of 15 different probes. For detection of the mature miRNA sequence, 5'DIG pre-labeled miRCURY LNA™ miRNA Detection Probes (Exiqon) were used at a final concentration of 10nM. Following probe reaction with anti-digoxigenin-POD (Roche) an amplification step was performed using the TSA Biotin System (NEN Life Science Products) which enhances the sensitivity to at least 5- to 10-fold. Hybridization temperatures for the different probes had been established in previous experiments. Probes were grouped in 4 sets and hybridized at 40°C, 50°C, 55°C or 65°C (for probe sequence and hybridization temperatures see Additional file 6). After ISH, slides were photographed in a light microscope (Leica DM-RXA2) equipped with a motorized stage, a Leica electronic focusing system, a Hitachi CCD camera and a PC-based controller that drives stage and camera.

## List of Abbreviations

BP: Biological Processes; DAVID: Database for Annotation, Visualization, and Integrated Discovery; D-V: Dorsal-Ventral; E: Embryonic day; GCL: Ganglion Cell Layer; GO: Gene Ontology; INBL: Inner Neuroblastic Layer; INL: Inner Nuclear Layer; IPE: Iris Pigmented Epithelium; ISH: *in situ *hybridization; KEGG: Kyoto Encyclopedia of Genes and Genomes; LESCs: Limbal Epithelial Stem Cells; MEV: Multi-Experiment Viewer; miRNA: microRNA; ONBL: Outer Neuroblastic Layer; ONL: Outer Nuclear Layer; P: Postnatal day; PHOT: Photoreceptors; RPE: Retinal Pigment Epithelium.

## Competing interests

The authors declare that they have no competing interests.

## Authors' contributions

MK designed and carried out the microarray experiment, participated in the analysis of the ISH data and the preparation of the database and drafted the manuscript. IP carried out the ISH experiments and participated in the analysis of the ISH data, the preparation of the database and the drafting of the manuscript. VAG performed the Gene Ontology analysis and participated in the interpretation of the microarray data. MB and RV carried out the ISH experiments. GL designed the database. PD participated in the design of the study and revised the draft of the manuscript. SB conceived the study and participated in its design and coordination, MK and SB wrote the final version of the manuscript. All authors read and approved the final manuscript.

## Supplementary Material

Additional file 1 **miRNA profiling of the adult mouse retina, RPE, cornea and lens**. Heat-map of two-way hierarchical clustering analysis for 285 miRNAs with Delta Log2 median ratio ΔLMR ≥ 0.5 in at least one of the samples (panel A). Only miRNAs with values across all samples were considered for the analysis. Colors indicate relative expression compared to the mean for each miRNA in the entire eye: red color represents an expression level above mean, green color represents expression lower than the mean. The complete set of nodes can be seen. The 5 clusters (A-E) obtained at the five-node level are delimited by green brackets. The 25 miRNA sub-clusters selected are highlighted with blue lines. The clustering of the tissues analyzed based on their miRNA content is shown by the horizontal dendrogram in panel B. sc: sub-cluster. Click here for file

Additional file 2 **Complete dataset of the Gene Ontology analysis**. The worksheet 'gsea_results_intersection' includes all the enriched GO/KEGG terms in biological processes and pathways for each of the predicted targets of the miRNAs present within each selected sub-cluster. The worksheet 'gsea_results_occurencies' summarizes the occurrence of each GO/KEGG term among the miRNA members of each selected node. The number of the miRNA members of each sub-cluster considered in the analysis is given in the last column. Click here for file

Additional file 3 **Gene Ontology analysis of the predicted targets of selected sub-clusters of miRNAs with comparable expression profiles**. Schematic representation of selected GO/KEGG categories relevant to eye development and function that were enriched among the targets of at least 70% of the miRNAs present within each sub-cluster. The miRNAs within each sub-cluster considered for functional analysis of their predicted targets are listed on the last column of the table. The tissues in which each cluster was predominantly expressed are listed in the column labeled "Expression". For each GO/KEGG category the percentage of representation of this term among the miRNAs analyzed is reported within the cell. The terms of BP Biological Processes and KEGG pathways that relate to similar cellular functions are arbitrarily coloured for readers' clarity. The exact reference numbers that correspond to the GO Biological Processes and KEGG pathways of the table are the following: cell adhesion (GO:0007155); axonogenesis (GO:0007409); morphogenesis of a branching structure (GO:0001763); modification-dependent macromolecule catabolic process (GO:0043632); epithelium development (GO:0060429); transmission of nerve impulse (GO:0019226); cell migration (GO:0016477); cell motion (GO:0006928); neuron development (GO:0048666); neuron differentiation (GO:0030182); neuron projection development (GO:0031175); pattern specification process (GO:0007389); cell-cell signaling (GO:0007267); synaptic transmission (GO:0007268); vesicle-mediated transport (GO:0016192); vasculature development (GO:0001944); Wnt signaling pathway (GO:0016055); regulation of actin cytoskeleton (KEGG: mmu04810); Adherens junction (KEGG: mmu04520); Axon guidance (KEGG: mmu04360); Focal adhesion (KEGG: mmu04510); Glioma (KEGG: mmu05214); GnRH signaling pathway (KEGG: mmu04912); Melanogenesis (KEGG: mmu04916); Melanoma (KEGG: mmu05218); Neurotrophin signaling pathway (KEGG: mmu04722); Wnt signaling pathway (KEGG: mmu04310); MAPK signaling pathway (KEGG: mmu04010); TGF-beta signaling pathway (KEGG: mmu04350). Abbreviations are, cor: cornea; ret: retina; RPE: retinal pigment epithelium. Click here for file

Additional file 4 **Detailed annotation of the miRNA expression patterns obtained at all developmental stages analyzed**. The table presents the overall pattern of expression (*e.g*. ubiquitous, ubiquitous with pattern, regional, not detected) at each developmental stage and a brief annotation of the structures that were positive for miRNA expression at each stage. Abbreviations are as follows: *Developmental stage: *E = Embryonic day; P = Postnatal day. *Overall signal: *nd = not detected; reg/1-2-3 = regional; ubi/1-2-3 = ubiquitous; uwp/1-2-3 = ubiquitous with pattern; 1-2-3 = signal intensity level *Anatomical structures: *BR = Brain; CB = Ciliary body; EOM = Extraocular muscles; GCL = Ganglion Cell Layer; INBL = Inner Neuroblastic Layer; INL = Inner Nuclear Layer; IR = Iris; OLF = Olfactory epithelium; ONBL = Outer Neuroblastic Layer; ONL = Outer Nuclear Layer; PHOT = Photoreceptors; RPE = Retinal Pigment Epithelium. Click here for file

Additional file 5 **Complete list of differential expression values [Log2 median ratios and Delta Log2 median ratios between each sample group (average) versus EYE1 sample]**. This page contains an expression matrix for all 9 samples analyzed by microarray. Only miRNAs with 9 values (no 'NA's accepted) across samples have been included in the analysis (see column named 'count'). In column 'M' to 'P' the difference in Log2 median ratios (ΔLMR) between each sample group (average) has been calculated to the 'entire eye' common reference sample named 'EYE1'. The miRNAs have been sorted on the basis of the values in column 'Q' with the highest differential expression on top (see column named MAX). The acronym 'NA' indicates miRNAs that did not yield reliable read-outs (see Methods for further details). The numbers are all log2(Hy3/Hy5) ratios. Click here for file

Additional file 6 **Probes used for RNA *in situ *hybridization**. The table summarizes information on probe sequence, miRBase accession number and hybridization (Thyb) temperatures. Click here for file
