# Supplementary material for: miRNeye: a microRNA expression atlas of the mouse eye
Source: BMC Genomics. 2010 Dec 20;11:715. doi: 10.1186/1471-2164-11-715 (PMC3018480; doi:10.1186/1471-2164-11-715)
Supplement: Additional file 3 — Gene Ontology analysis of the predicted targets of selected sub-clusters of miRNAs with comparable expression profiles. Schematic representation of selected GO/KEGG categories relevant to eye development and function that were enriched among the targets of at least 70% of the miRNAs present within each sub-cluster. The miRNAs within each sub-cluster considered for functional analysis of their predicted targets are listed on the last column of the table. The tissues in which each cluster was predominantly expressed are listed in the column labeled "Expression". For each GO/KEGG category the percentage of representation of this term among the miRNAs analyzed is reported within the cell. The terms of BP Biological Processes and KEGG pathways that relate to similar cellular functions are arbitrarily coloured for readers' clarity. The exact reference numbers that correspond to the GO Biological Processes and KEGG pathways of the table are the following: cell adhesion (GO:0007155); axonogenesis (GO:0007409); morphogenesis of a branching structure (GO:0001763); modification-dependent macromolecule catabolic process (GO:0043632); epithelium development (GO:0060429); transmission of nerve impulse (GO:0019226); cell migration (GO:0016477); cell motion (GO:0006928); neuron development (GO:0048666); neuron differentiation (GO:0030182); neuron projection development (GO:0031175); pattern specification process (GO:0007389); cell-cell signaling (GO:0007267); synaptic transmission (GO:0007268); vesicle-mediated transport (GO:0016192); vasculature development (GO:0001944); Wnt signaling pathway (GO:0016055); regulation of actin cytoskeleton (KEGG: mmu04810); Adherens junction (KEGG: mmu04520); Axon guidance (KEGG: mmu04360); Focal adhesion (KEGG: mmu04510); Glioma (KEGG: mmu05214); GnRH signaling pathway (KEGG: mmu04912); Melanogenesis (KEGG: mmu04916); Melanoma (KEGG: mmu05218); Neurotrophin signaling pathway (KEGG: mmu04722); Wnt signaling pathway (KEGG: mmu04310); MAPK signaling [file 1471-2164-11-715-S3.PDF]

Sub-cluster	Expression	GO Biological Processes																	KEGG pathways												miRNA members
		neuron differentiation	neuron development	transmission of nerve impulse	synaptic transmission	neuron projection development	axonogenesis	pattern specification process	vesicle-mediated transport	modification-dependent macromolecule catabolic process	cell motion	cell migration	morphogenesis of a branching structure	vasculature development	epithelium development	cell-cell signaling	cell adhesion	Wnt signaling pathway	Neurotrophin signaling pathway	Glioma	Melanoma	Melanogenesis	Axon guidance	Regulation of actin cytoskeleton	Focal adhesion	Adherens junction	Wnt signaling pathway	MAPK signaling pathway	TGF-beta signaling pathway	GnRH signaling pathway	
**sc_1**	RPE, ret	x	x	x	x	x	x	x	x	x	x	x	x	x	x	x	100	x	x	x	x	x	x	x	x	x	x	x	x	x	miR-126-5p, miR-451, miR-652
**sc_2**	RPE	83	x	x	83	x	x	x	100	x	x	x	x	x	x	x	x	x	100	83	x	x	100	x	100	83	x	83	x	x	miR-145, miR-143, miR-146b, let-7c, miR-466b-3-3p, miR-466a-3p/ miR-466b-3p/miR-466c-3p/ miR-466e-3p
**sc_3**	RPE, cor	88	88	x	x	88	x	x	x	x	88	88	x	x	x	75	x	x	75	x	x	88	100	88	88	88	75	100	x	x	let-7b, miR-142-5p, miR-142-3p, miR-297a, miR-574-5p, miR-706, miR-497, miR-669a
**sc_4**	RPE	x	x	x	x	x	x		x	x	x	x	x	x	x	x	x	x	78	x	x	x	89	x	78	x	89	89	x	x	miR-223, miR-883a-5p, miR-214, miR-467e, miR-1196, miR-150, miR-877, miR-125b-5p, miR-665
**sc_5**	ret	75	75	75	x	75	x	88	x	x	75	88	x	75	x	x	x	x	100	88	x	x	100	88	75	x	x	100	x	x	miR-186, miR-361, miR-181b, miR-181d, miR-708, miR-331-3p, miR-301a, miR-181c
**sc_6**	ret, RPE	x	x	x	x	x	x	x	x	x	x	x	x	75	x	x	x	x	x	x	x	x	88	x	x	x	x	75	x	x	miR-328, miR-532-5p, miR-185, miR-124, miR-151-5p, miR-140, miR-339-5p, miR-211
**sc_7**	ret	x	83	x	x	x	x	x	x	x	x	x	x	x	x	x	x	x	83	x	x	x	83	x	83	x	83	100	x	x	miR-541, miR-377, miR-136, miR-434-3p, miR-7b, miR-101b
**sc_8**	ret	x	x	x	x	x	x	x	x	x	x	x	x	x	x	x	x	x	x	x	x	x	75	x	88	x	x	75	x	x	miR-425, miR-219, miR-598, miR-495, miR-369-3p, miR-335-5p, miR-376a, miR-484
**sc_9**	ret	x	80	x	x	x	x	80	x	x	x	x	x	x	x	x	x	x	80	x	x	x	100	100	100	80	100	100	80	x	miR-190b, miR-194, miR-129-3p, miR-148b, miR-153
**sc_10**	ret	x	x	x	x	x	x	x	x	x	x	x	x	x	x	x	x	x	100	88	x	x	100	x	88	x	100	88	x	x	miR-9, miR-345-5p, let-7g, miR-138, miR-500, miR-132, miR-29c, miR-197
**sc_11**	ret	70	70	x	x	70	x	x	x	x	80	80	x	70	x	x	x	x	90	70	x	x	90	80	80	x	x	90	x	x	miR-183, miR-362-3p, miR-96, miR-30e, miR-212, miR-191, miR-326, miR-30a, miR-101a, miR-182
**sc_12**	ret, cor, RPE	x	x	x	x	x	x	x	x	x	x	x	x	x	x	x	x	x	x	x	x	x	83	x	x	x	x	100	x	x	miR-434-5p, miR-106b, miR-127, miR-29b, miR-677, miR-29a
**sc_13**	ret, RPE	80	70	x	x	x	x	x	100	90	x	x	x	x	x	x	x	80	90	90	100	x	100	x	100	70	80	x	x	x	miR-30d, miR-30c, miR-144, miR-30b, miR-107, miR-103, miR-98, let-7d, let-7f, let-7a
**sc_14**	ret, RPE	100	100	x	x	88	75	75	x	x	x	x	x	75	x	x	x	x	88	75	x	x	100	75	100	x	75	88	x	x	miR-26a, miR-26b, miR-805, miR-1187, miR-34a, miR-423-5p, let-7i, miR-378
**sc_15**	ret, cor	x	x	x	x	70	x	x	70	x	70	x	x	x	x	x	x	x	80	80	x	x	70	70	80	70	70	80	x	80	miR-16, miR-411, miR-379, miR-15a, miR-685, miR-674, miR-25, miR-15b, miR-193, miR-21
**sc_16**	ret, lens	73	73	x	x	x	x	x	x	x	x	x	x	x	x	73	x	x	x	73	x	x	82	x	82	73	73	91	x	x	miR-181a, miR-7a, miR-872, miR-341, miR-128, miR-384-5p, miR-340-5p, miR-376b, miR-192, miR-382, miR-129-5p, miR-384-3p
**sc_17**	lens, ret	78	100	x	x	78	78	x	x	x	78	x	x	x	x	x	x	x	x	x	x	78	100	73	89	x	78	78	x	x	miR-218, miR-374, miR-34b-5p, miR-465b-5p, miR-582-5p, miR-499, miR-130b, miR-300, miR-375
**sc_18**	lens, cor	x	78	x	x	x	x	x	x	x	x	x	x	x	x	x	x	x	x	78	x	x	78	x	78	x	x	x	x	x	miR-93, miR-130a, miR-184, miR-17, miR-20a, miR-20b, miR-503, miR-31, miR-668
**sc_19**	lens	x	x	x	x	x	x	75	75	x	x	x	75	x	x	75	x	x	x	x	x	x	100	x	75	x	75	100	x	x	miR-542-3p, miR-770-3p, miR-494, miR-705
**sc_20**	lens, cor	80	x	x	x	x	x	x	x	x	x	80	x	x	x	x	x	x	x	x	x	x	80	x	x	80	100	x	x	x	miR-742, miR-290-5p, miR-470, miR-207, miR-720
**sc_21**	lens, cor	x	x	x	x	x	x	x	x	x	x	x	x	x	x	x	x	x	x	x	x	x	x	x	x	x	71	x	x	x	miR-582-3p, miR-675-5p, miR-667, miR-673-3p, miR-804, miR-874, miR-551b
**sc_22**	lens, RPE	80	80	x	x	80	80	x	100	x	80	x	x	x	x	x	x	x	80	x	x	x	80	80	x	x	x	80	x	x	miR-206, miR-125b-3p, miR-882, miR-204, miR-713
**sc_23**	lens	x	x	x	x	75	x	x	x	x	x	x	x	x	x	x	x	x	75	x	x	x	75	x	x	x	x	88	x	x	miR-325, miR-693-5p, miR-133a, miR-365, miR-883b-5p, miR-1, miR-133b, miR-710
**sc_24**	cor, RPE	83	x	x	x	x	x	x	x	x	x	x	x	x	75	x	x	x	75	x	x	x	92	92	92	83	92	92	x	x	miR-222, miR-27b, miR-322, miR-221, miR-490, miR-24, miR-199a-3p/miR-199b, miR-199a-5p, miR-23b, miR-23a, miR-22, miR-27a
**sc_25**	cor	88	x	x	x	x	x	75	88	75	88	75	88	75	x	x	x	x	75	75	x	x	100	88	88	100	88	100	x	88	miR-205, miR-200b, miR-429, miR-200c, miR-141, miR-200a, miR-203, miR-148a
